# Random Dots

**Published:** 1899-10

**Authors:** F. S. Caspar

**Affiliations:** Austin, Texas


### Dental Department.

F. S. CASPAR, D. D. S., AUSTIN, TEXAS.

### Random Dots.

DR. R. G. GUNN, a young dentist of Springfield, Ill., was robbed and had his skull fractured by footpads, July 28, 1899.—(*Exchange.*)

These two "K"lamaties could not happen to a Texas dentist. The first, because if he has any money left after paying his regular State, county and city taxes, the occupation tax takes the balance; secondly, if his head is ripe enough to be cracked (or "fractured" is a more surgical expression), he won't come to Texas. And Mr. Footpad knows it. See!

